# MHC-Linked Syngeneic Developmental Preference in Thymic Lobes Colonized with Bone Marrow Cells: A Mathematical model

**DOI:** 10.1155/1998/65943

**Published:** 1998

**Authors:** Ramit Mehr, Alan S. Perelson, Ayala Sharp, Lee Segel, Amiela Globerson

**Affiliations:** 1 Dept. of Molecular Biology Princeton University Princeton New Jersey 08544 USA; 2 Theoretical Division Los Alamos National Laboratory Los Alamos New Mexico USA; 3 Department of Molecular Cell Biology The Weizmann Institute of Science Rehovot 76100 Israel; 4 Department of Applied Mathematics and Computer Science The Weizmann Institute of Science Rehovot 76100 Israel; 5 Department of Immunology The Weizmann Institute of Science Rehovot 76100 Israel

**Keywords:** Thymus, T-cell development, mathematical model, MHC, simulations, syngeneic preference

## Abstract

Reconstitution of the T-cell compartment after bone marrow transplantation depends on successful colonization of the thymus by bone-marrow-derived progenitor cells. Recent studies compared the development of syngeneic and allogeneic bone-marrow-derived cells in cocultures with lymphoid-depleted fetal thymus explants, leading to the discovery of MHC-linked syngeneic developmental preference (SDP) in the thymus. To determine the nature of cell interactions among the bone marrow and thymic elements that might underlie SDP, we analyzed this phenomenon by mathematical modeling. The results indicate that syngeneic mature T cells, responsible for inducing this preference, probably interfere both with the seeding of allogeneic bone-marrow-derived thymocyte progenitors in the thymic stroma and with their subsequent proliferation. In addition, the possibility of augmented death among the developing allogeneic thymocytes cannot be ruled out.


*Developmental Immunology*, 1998, Vol. 5, pp. 303–318
Reprints available directly from the publisher
Photocopying permitted by license only

© 1998 OPA (Overseas Publishers Association)
N.V. Published by license under the
Harwood Academic Publishers imprint,
part of the Gordon and Breach Publishing Group
Printed in Malaysia

### MHC-Linked Syngeneic Developmental Preference in Thymic Lobes Colonized with Bone Marrow Cells: A Mathematical model

RAMIT MEHR[a]*, ALAN S. PERELSON[b], AYALA SHARP[c], LEE SEGEL[d] and AMIELA GLOBERSON[e]

[a]*Dept. of Molecular Biology, Princeton University, Princeton, New Jersey 08544;* [b]*Theoretical Division, Los Alamos National Laboratory, Los Alamos, New Mexico;* [c]*Department of Molecular Cell Biology;* [d]*Department of Applied Mathematics and Computer Science, and* [e]*Department of Immunology* the Weizmann Institute of Science, Rehovot 76100, Israel

*(Received 6 January 1997; In final form 29 July 1997; Accepted 30 September 1997)*

Reconstitution of the T-cell compartment after bone marrow transplantation depends on successful colonization of the thymus by bone-marrow-derived progenitor cells. Recent studies compared the development of syngeneic and allogeneic bone-marrow-derived cells in co-cultures with lymphoid-depleted fetal thymus explants, leading to the discovery of MHC-linked syngeneic developmental preference (SDP) in the thymus. To determine the nature of cell interactions among the bone marrow and thymic elements that might underlie SDP, we analyzed this phenomenon by mathematical modeling. The results indicate that syngeneic mature T cells, responsible for inducing this preference, probably interfere both with the seeding of allogeneic bone-marrow-derived thymocyte progenitors in the thymic stroma and with their subsequent proliferation. In addition, the possibility of augmented death among the developing allogeneic thymocytes cannot be ruled out.

*Keywords:* Thymus, T-cell development, mathematical model, MHC, simulations, syngeneic preference

### INTRODUCTION

In bone marrow (BM) transplantations, the reconstitution of the T-cell compartment by donor BM-derived progenitor cells depends on their successful colonization of the host thymus, which in turn depends on their histocompatibility with respect to the host thymus, and on the dose of BM cells inoculated (Reisner and Martelli, 1995). For these reasons, the role of major histocompatibility complex (MHC) molecules in thymocyte development has been studied extensively (von Boehmer, 1991; von Boehmer and Kisielow, 1990; Kisielow and von Boehmer, 1990; Blackman et al., 1990). Recent experimental studies (Eren et al., 1989; Sharp et al., 1991, 1995; Fridkis-Hareli et al., 1993) suggest that, in addition to their role in selection processes, which occur relatively late in thymocyte development (in the late double positive and single positive stages), MHC molecules may also play a role in the very early

*Corresponding author. Tel.: 609-258-3001, Fax: 609-258-2205, E-Mail: ramit@princeton.edu.

events that occur when BM-derived T-cell progenitors, which do not express the TCR or functional T-cell markers, first enter the thymus. The term "MHC-linked syngeneic developmental preference (SDP)" was coined to describe the competitive advantage that syngeneic bone marrow (BM) cells have over allogeneic cells when colonizing the thymus under these conditions. ("Syngeneic" cells are defined as having the same MHC type as the thymic stroma being colonized, and "allogeneic" cells are defined as having a different MHC type from the thymic stroma.) In the present study, we use mathematical modeling of thymocyte dynamics to screen hypotheses concerning possible mechanisms underlying this phenomenon.

Thymocyte development begins with the seeding of progenitor cells onto specialized "seeding niches" (Shortman et al., 1990) in the thymic cortex. Our *in vitro* studies examined the ability of syngeneic and allogeneic BM cells to colonize lymphoid-depleted fetal thymuses (FT). Allogeneic BM cells on their own were found to be capable of reconstituting the FT to similar levels as syngeneic BM cells. However, when allogeneic BM cells were mixed (at a 1:1 ratio) with syngeneic BM cells, a developmental preference of the syngeneic BM cells was observed: More than 80% of the developing thymocytes originated from the syngeneic BM (Eren et al., 1989; Sharp et al., 1991). Genetic mapping experiments (Eren et al., 1989) suggested that the MHC class II (I-E and I-A) region may be important in determining SDP; hence, we refer to this phenomenon as "MHC-linked." Experiments of sequential seeding, in which BM cells from one source were seeded first, and 24 hr later cells from another source were added to the culture and incubated for two additional days, show that the major impact of MHC-linked competition was related to events occurring in the first 24 hr of seeding (Sharp et al., 1991)—an earlier stage than previously described for the involvement of MHC molecules in selection processes in the thymus.

Further studies were aimed at identifying the cells involved in the induction of SDP (Sharp et al., 1995). When BM cells were depleted of mature T cells (i.e., of Thy1$^+$ or CD3$^+$ cells), no SDP was observed, suggesting that mature T cells in the seeded inoculum, especially those from the syngeneic origin, may play a role in determining SDP. The effects of mature T cells were then examined by the addition of peripheral blood lymphocytes (PBL) to the Thy1$^-$ BM cells (of syngeneic or allogeneic origin, or mixtures of both) that were seeded. The amount in the inoculum was 5-10% of the BM cells, in accordance with the normal proportion of lymphocytes in the intact BM of these mice. The PBL added were either syngeneic or semiallogeneic; the latter term refers to cells expressing both similar and different MHC haplotypes, with respect to the FT stroma. Thymocytes derived from the syngeneic BM, and the FT stroma, expressed one type of Thy1 marker (Thy1.1$^+$), whereas thymocytes derived from the allogeneic BM were Thy1.2$^+$. The PBL were from donors expressing both cell-surface markers, Thy1.1 and Thy1.2. Thus, the contribution of PBL themselves to the total number of cells obtained in the cultures could be distinguished from the contribution of the seeded progenitor cells, which originated from either Thy1.1$^+$Thy1.2$^-$ or Thy-1.1$^-$Thy1.2$^+$ genotypes.

It was found that the addition of PBL, whether syngeneic or semiallogeneic, reduced the total cell numbers obtained from the cultures, even when only syngeneic progenitors were seeded (Sharp et al., 1995). However, the identity of the PBL had a marked effect on the ratios of syngeneic and allogeneic BM-derived cells in the reconstituted FT. Allogeneic cells failed to develop in the presence of syngeneic PBL, and a limited level of allogeneic BM-derived cells was found in cocultures with semiallogeneic PBL (Sharp et al., 1995). Experimental results are summarized in what follows.

The aim of the present study was to elucidate the mechanism of MHC-linked SDP, through mathematical modeling and analysis of competing hypotheses concerning the cause(s) of this phenomenon. We studied whether SDP is determined primarily during the seeding stage or also later in thymocyte development, and whether the involvement of mature T cells is limited to syngeneic T cells.

### HYPOTHESES AND MODEL

Several hypotheses may be considered as explanations of the SDP phenomenon. The results of sequential seeding suggest that the effects are exerted early, possibly during the seeding stage. We considered the following alternative hypotheses concerning the involvement of syngeneic mature T cells in seeding. (1) A negative effect of syngeneic mature T cells on allogeneic progenitor interaction with the stroma, for examples interference with their homing to thymic seeding niches, or subsequent proliferation. These effects may be due to competition for cell-cell interactions. There also may be actual killing of allogeneic progenitors by syngeneic mature cells, or augmentation of programmed cell death. (2) A positive effect of syngeneic mature T cells on syngeneic progenitor interaction with the thymic stroma. These two hypotheses are not mutually exclusive.

The present analysis formulates the preceding hypotheses mathematically, in order to evaluate the possible contribution of each of the proposed effects. The type of general model that can be developed at this stage cannot answer questions about the detailed mechanisms through which the effects are exerted. However, it may indicate whether the experimental findings can be reconstructed in a simulation under various hypotheses concerning the stage of development at which the effects may be exerted, and whether the effects are positive or negative. We base our model on the following principles.

(a) We ignore possible changes in the thymic stroma due to interactions with developing T cells (Francz et al., 1992), and deal only with unidirectional effects of the stroma on the lymphoid elements. The latter are expressed in our model only implicitly, through modification of thymocyte replication parameters, and there is no separate equation for stromal cells.

(b) We represent the development of each BM-derived thymocyte population by a three-compartment model: The newly seeded cell compartment (the number of cells in this compartment is represented by the variable $N$) that contains immature thymocytes that were recently seeded in the thymus; the differentiation/proliferation or growing compartment (represented by the variable $G$) containing all developing thymocytes except the newly seeded ones; and the BM-derived mature T-cell compartment, represented by $M$. The scheme on which the model is based is presented for one population in Fig. 1. The newly seeded cell compartment is considered separately from the rest of thymocyte development for two reasons. First, the behavior of cells in this compartment is indeed different from that of later thymocytes: They compete not on all thymic resources, but on specific "seeding niches," the number of which is limited ($\approx$100) (Shortman et al., 1990; Mehr et al., 1994). Second, the experiments suggest that MHC-linked SDP is already exerted at this stage, so that we separate (in the model) the possible effects of mature T cells on the seeding process itself from their effects on subsequent cell division and differentiation. The mature T cells derived from the BM progenitors are not likely to become effective in the first wave of colonization followed in these experiments. However, we include them in the model (compartment $M$) for the sake of completeness, and show later that, indeed, their effect is negligible. We also consider separately the number of mature cells from the peripheral blood lymphocyte (PBL) source, $P$. This separation enables the comparison of our results to experiments; however, we assume that mature cells from both sources ($M$ and $P$) have the same cell division and death rates, and the same effects on other cell compartments.

(c) We assume that cells of all compartments except the newly seeded cell compartment compete for space and resources within the thymus (Metcalf, 1963).

(d) Support of progenitor cells by the stroma is assumed to be the same for all progenitor cell types. Hence, the replication parameters for all types of cells in the newly seeded cell compartment are the same in our model.

The model equations derived from these principles are presented in the appendix.

### COMPETITIVE SEEDING

The results of competitive seeding experiments were expressed in terms of the relative reconstitution ratio, defined by

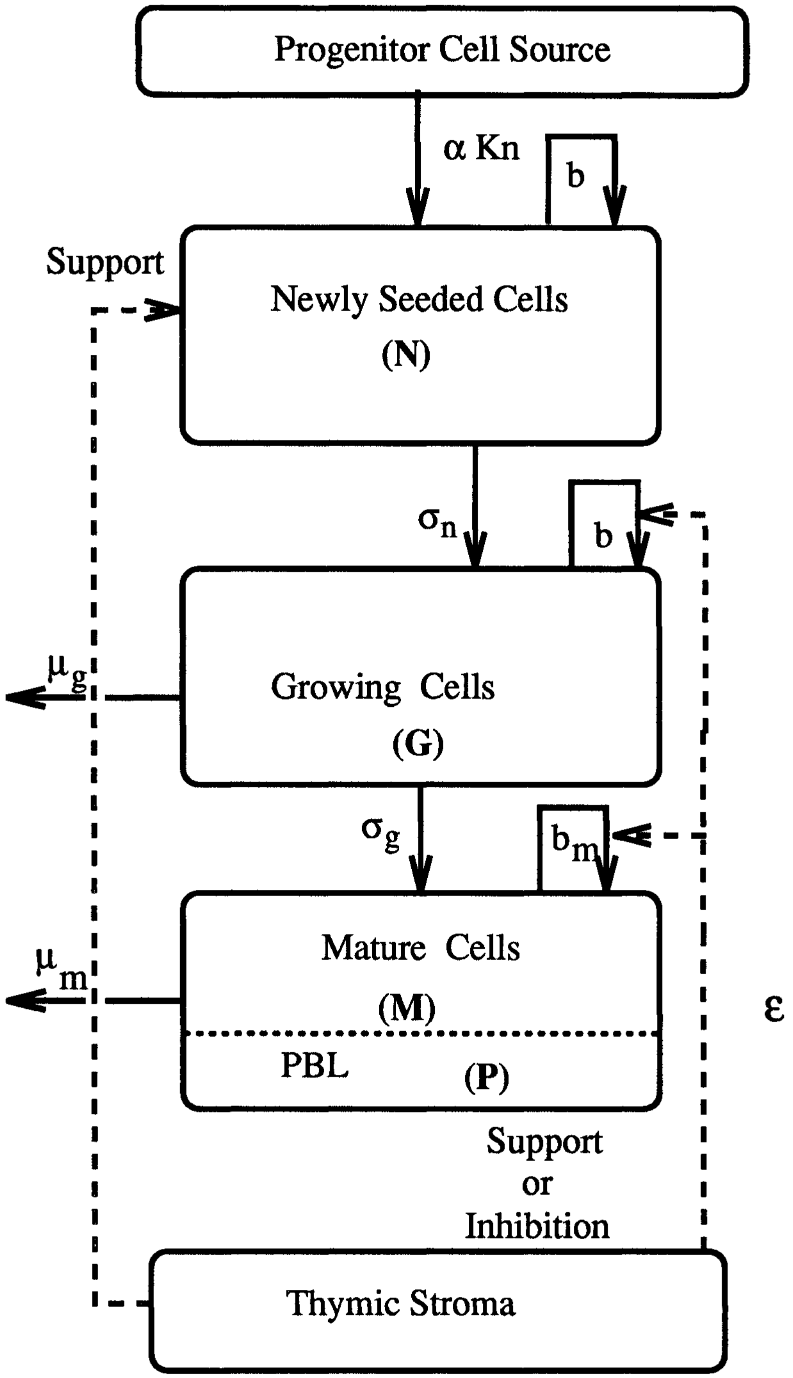

FIGURE 1 The cell compartments and their interactions for the case of one population (syngeneic or allogeneic) only. Solid arrows indicate differentiation of cells from one compartment to the next, proliferation, or death; dashed arrows indicate the effects of thymic stroma, which is not included explicitly in our equations. Parameters indicate the rates of the various processes.

$$RR \equiv \frac{\text{syn}}{\text{syn} + \text{allo}} \times 100 \qquad (1)$$

where "syn" denotes the percent of BM-derived syngeneic cells, and "allo" denotes the percent of BM-derived allogeneic cells obtained in the cultures. The sum of syngeneic and allogeneic cell percents is less than 100% because there is always a fraction of cells that are negative to both markers. In our simulations, $RR$ is defined as

$$RR = \frac{N_s + G_s + M_s}{N_s + G_s + M_s + N_a + G_a + {}_a} \times 100 \qquad (2)$$

where subscript $s$ denotes syngeneic populations and $a$ denotes allogeneic populations, as defined in Fig. 2.

In order to compare our results to experimental data, we consider three experimental protocols. In competitive seeding (protocol "$C$"), both populations are seeded at the same time at a 1:1 ratio, but the syngeneic and allogeneic seeding rates, $\alpha_s$ and $\alpha_a$, differ from zero only for the first 3 days, after which seeding is terminated. The other two are sequential seeding protocols: one (protocol "$S \rightarrow A$") in which syngeneic cells are seeded first ($\alpha_s > 0$, $\alpha_a = 0$ in the

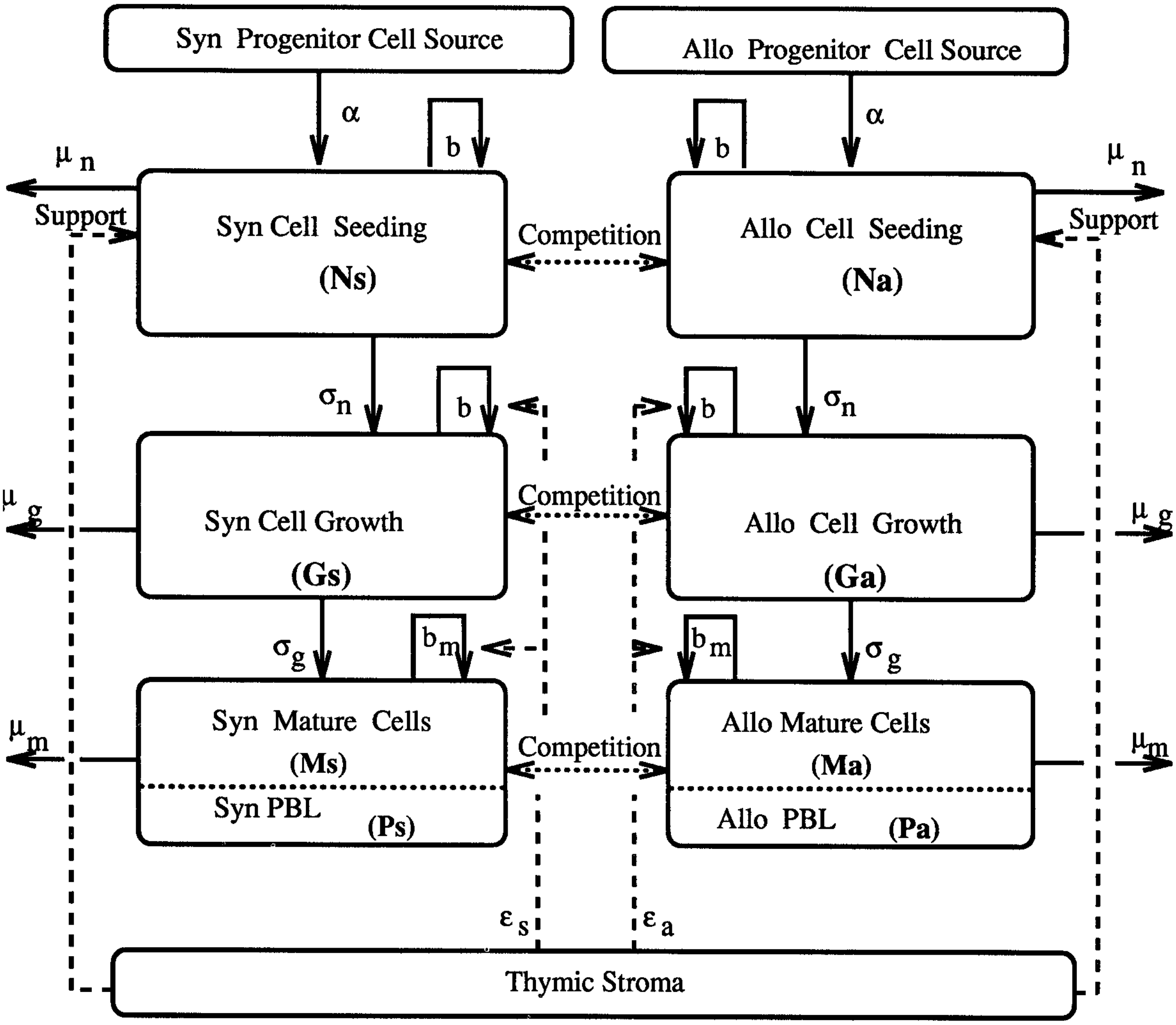

FIGURE 2 The cell compartments and their interactions for the case of two populations, syngeneic (Syn) and allogeneic (Allo).

TABLE I Experimental Relative Reconstitution Ratios[a]

| Experiment | | $RR_c$ | $RR_{S\to A}$ | $RR_{A\to S}$ |
|---|---|---|---|---|
| 1 Unseparated BM | No PBL | 82 ± 14 | 80 ± 10 | 40 ± 10 |
| 2 $Thy1^-$ BM | No PBL | 54 ± 6 | ND | ND |
| 3 $Thy1^-$ BM | SYN PBL | 88 ± 6 | ND | ND |
| 4 $Thy1^-$ BM | SA PBL | 67 ± 8 | ND | ND |

[a]Relative reconstitution ratios as defined in Eq. (1), from competitive ($RR_c$) or sequential seeding experiments ($RR_{S\to A}$ and $RR_{A\to S}$). Results are reproduced or averaged from (Eren et al., 1989; Sharp et al., 1991, 1995). Cells were seeded on the FT, as described in the text, and then the FT were cultured for 11 days, after which the resulting thymocytes were harvested. When PBL were used, they were added as 10% of the seeded BM inoculum. ND: not done.

first day or two, and $\alpha_a > 0$, $\alpha_s = 0$ in the next 2 days), and another (protocol "$A \rightarrow S$") in which allogeneic cells are seeded first. Accordingly, we denote the relative reconstitution ratios for these protocols by $RR_c$, $RR_{S\to A}$, and $RR_{A\to S}$.

If all parameters were the same for both cell types, then our model predicts that the results of the three protocols would be symmetric, that is: (1) $RR_c = 50$, that is, a 1:1 syngeneic:allogeneic ratio; (2) $RR_{S\to A} > 50$, only because cells seeded first have an opportunity to occupy more seeding niches; and (3) $RR_{A\to S} = (100 - RR_{S\to A})$, because $RR$ is defined as the percentage of *syngeneic* donor-derived thymocytes in the cocultures, and hence it would be smaller than 50 if allogeneic cells are seeded first.

In contrast to this prediction, in the experiments, the results are *not* symmetric, unless there are no mature T cells present. Table I shows the range of values from several experiments (Eren et al., 1989; Sharp et al., 1995). Unseparated BM, which contain up to 10% mature T cells, were seeded in all three protocols (Table I, line 1). It is evident that $RR_{A\to S} > (100 - RR_{S\to A})$, meaning that although when allogeneic cells are seeded first, they gain an advantage over syngeneic cells, this advantage is not comparable to the advantage syngeneic cells have when seeded first or even in a 1:1 mixture with allogeneic cells. This is what we refer to as an "asymmetric" result, which indicates that there are differences between thymic colonization by syngeneic and allogeneic progenitor cells. When mature T cells are removed by depleting the BM of $Thy1^+$ cells, SDP is not observed (Table I, line 2), but it is restored by the addition of syngeneic or semiallogeneic PBL (Table I, lines 3-4). Only protocol "$C$" was used in the experiments utilizing $Thy1^-$ BM cells.

Our goal is to elucidate those interactions that are important in generating the phenomenon of MHC-linked SDP. Attempting a complete survey of parameter space is not feasible. Instead, we determine which hypotheses can, and which cannot, account for the experimental results when implemented in computer simulations of our model using a reasonable combination of parameter values. Unsuccessful hypotheses may (at least tentatively) be excluded from further experimental examination. The following is a summary of the results of these simulations.

### Competitive Seeding in the Absence of Mature Cells

We solved the two-population-model equations (given in the appendix) using the parameters given in Table II. The initial conditions for the simulations of $Thy1^-$ cell seeding were based on the following data. In each experiment, a total $6 \times 10^4$ BM cells were seeded. PBL, when added, always constituted of 10% of the seeded cells, that is, 6000 cells. In sequential seeding, the 4200 mature T cells assumed to be included in the *second* BM inoculum were added at $t = 48$ hr.

The values of $\alpha$, $b$, and $\mu_n$ were determined in previous studies (Eren et al., 1990; Mehr et al., 1993, 1994). Simulating the experiments of competitive seeding in which there were initially no mature T cells in the BM enabled us to check our choice of parameters, and determine the values of $K$, $\epsilon_s$, $\epsilon_a$, and $\mu_g$. In order to obtain a total number of cells similar to

TABLE II Simulation Parameters[a]

| Simulation | Parameter | Value |
|---|---|---|
| All simulations | $K$ | 150,000 |
| | $K_n$ | 100 |
| | $K_{m_s}$ | 3,000 |
| | $K_{m_a}$ | 6,000 |
| | $b$ | 0.05 |
| | $\mu_n$ | 0 |
| | $\mu_g$ | 0.01 |
| | $b_m$ | 0.005 |
| | $\mu_m$ | 0 |
| | $\delta_n$ | 0.01 |
| | $\epsilon_s$ | 0 |
| | $\epsilon_a$ | −0.013 |
| | $\lambda_s$ | 0 |
| | $\lambda$ | 0 |
| No $G \rightarrow M$ differentiation | $\delta_g$ | 0 |
| With $G \rightarrow M$ differentiation | $\delta_g$ | 0.001 |
| | Rates of seeding[b] | |
| T−/C/P+/first 72 hr | | $\alpha_s = \alpha_a = 0.0081$ |
| T−/C/P−/first 72 hr | | $\alpha_s = \alpha_a = 0.009$ |
| T−/S→A/P+/first 72 hr | | $\alpha_s$ or $\alpha_a = 0.0161$ |
| T−/S→A/P−/first 72 hr | | $\alpha_s$ or $\alpha_a = 0.0179$ |
| All simulations/t > 72 hr | | $\alpha_s = \alpha_a = 0$ |

[a]The parameters for simulations of competition in the absence of externally added mature T cells. Mature T cells can appear in this situation only in the case in which the differentiation from the $G$ to the $M$ compartment is nonzero. The choice of parameter values is explained in the text.

The choice of the values for the rates of seeding, $\alpha_s$ and $\alpha_a$, was based on the following data. In unseparated BM of young adult mice, the fraction of thymocyte progenitors is about 0.002 (Eren et al., 1990). This was multiplied by the number of BM cells of each type seeded (the total was $6 \times 10^4$ cells in all cases), and divided by the time of seeding (3-4 days) to give an estimate for the seeding rate, as done previously (Mehr et al., 1994). The numbers were appropriately modified when the seeding of Thy1$^-$ BM cells was simulated. The type of simulation is denoted as follows. T−: Thy1$^-$ BM. P+: PBL were added; P− no PBL were added. The protocol of seeding is denoted as "*C*", "*S*→*A*" and so forth and the values of the seeding rates are nonzero only in the first 72 hr of seeding. These simulations included situations that were not examined experimentally, that is, sequential seeding of Thy1-depleted cells.

that obtained in the experiments, we chose the maximum value to be $K = 1.5 \times 10^5$ cells, which is reasonable for fetal thymuses after 10-11 days in organ culture, and a small death rate for growing cells, $\mu_g = 10^{-2}\ h^{-1}$. Larger values for $\mu_g$ (e.g., $10^{-1}\ h^{-1}$) result in total cell numbers that are orders of magnitude smaller than the experimental data. To achieve an even finer fit to experimental results, we chose $\epsilon_s = 0$ and $\epsilon_a = -0.013$, which provide the slight advantage syngeneic cells have in these conditions. Simulations using these parameters gave "nearly symmetrical" results, which are reported here both in terms of cell numbers and of $RR_c$ (Table III).

Cell numbers and $RR_c$ in the first three rows in Table III are comparable to those obtained in the corresponding experiments (Table I, line 2). The last two rows in Table III represent situations that were not examined in experiments, that is, sequential seeding of Thy1-depleted BM cells, and give the prediction of our model for these situations under the same hypothesis. Our model clearly predicts that the seeding stage is crucial: The population that is given an advantage of only 2 days—in which no more than one or two cell divisions may occur—in seeding, will eventually dominate, since the initial advantage enables it to occupy most of the seeding niches. These results support the conclusions of our previous studies (Mehr et al., 1993, 1994, 1995) that events in the seeding stage largely determine the outcome of thymic colonization.

TABLE III Simulations without, Mature Cells[a]

| Cells | Seeding protocol simulated[b] First | Second | SBM ($\times10^4$) | ABM ($\times10^4$) | $RR_c$ |
|---|---|---|---|---|---|
| 1 BM (Thyl$^-$) | SYN + ALLO | SYN + ALLO | 6.5 | 5.5 | 54 |
| 2 BM (Thyl$^-$) | SYN | SYN | 12 | 0 | 100 |
| 3 BM (Thyl$^-$) | ALLO | ALLO | 0 | 12 | 0 |
| 4 BM (Thyl$^-$) | SYN | ALLO | 11.8 | 0.2 | 98 |
| 5 BM (Thyl$^-$) | ALLO | SYN | 0.4 | 11.6 | 3 |

[a]Cell numbers and $RR_c$ values [relative reconstitution ratios of syngeneic to allogeneic BM-derived thymocytes, as defined in Eq. 2)], from simulations *without* mature T cells. Parameters for these simulations are given in Table II.
[b]SBM: Syngeneic BM-derived thymocytes; ABM: Allogeneic BM-derived thymocytes. When SYN+ALLO cells were seeded, it was always a 1:1 mixture. "First" and "Second" refer to the first and second seeding phases. The last two lines represent situations that were not examined experimentally, that is, sequential seeding of Thyl$^-$ BM cells without mature T cells (see text).

TABLE IV Simulations with PBL[a]

| Cells | Seeding protocol simulated First and Second | PBL | SBM ($\times10^4$) | ABM ($\times10^4$) | $RR_c$ |
|---|---|---|---|---|---|
| BM (Thyl$^-$) | SYN + ALLO | — | 6.5 | 5.5 | 53 |
| BM (Thyl$^-$) | SYN + ALLO | SYN | 5.2 | 4.4 | 53 |
| BM (Thyl$^-$) | SYN + ALLO | SA | 5.2 | 4.4 | 53 |
| BM (Thyl$^-$) | SYN + ALLO | ALLO | 5.2 | 4.4 | 53 |

Results of simulations with PBL—competition only (all $H_i^{jk} = 0$). Other details are as in Table III

### Seeding of Thyl$^-$ BM in the Presence of PBL

We repeated the simulations of seeding of Thyl$^-$ BM cells, this time assuming the presence of peripheral blood lymphocytes (PBL), in three possible combinations: only syngeneic PBL, only allogeneic PBL (not included in the experiments), and semi allogeneic PBL.

Since there is no evidence for mature T-cell death, we chose $\mu_m = 0$. Our choice of the value for $b_m$ is limited by the requirement that both the total cell numbers and PBL cell numbers remain compatible with experimental values. Assuming that PBL seeded onto FT lobes remain there, without growing ($b_m = 0$) or interacting with BM-derived thymocytes, gives results that are not significantly different from those of simulations without PBL. Moreover, assuming that PBL do divide in the thymus, and hence compete with BM-derived cells (i.e., setting $b_m > 0$), still gives similar results (data not shown). To match the observed value of total cell numbers, we had to take $0.002 < b_m < 0.01$. In this range, the $RR_c$ values still do not show the SDP evident in experiments: in "mixture seeding" simulations (Table IV, using $b_m = 0.005$) and we get $RR_c = 53$ whether any PBL were added or not. Hence, our first conclusion is that competition alone cannot account for the observed phenomena, and we proceeded to examine the effect of additional possible interactions.

### EFFECTS OF MATURE T CELLS ON LYMPHOCYTE DEVELOPMENT

To model the effects of mature T cells on the various parameters of thymocyte growth, we modify each parameter at a time, by multiplying it by a saturating function of the number of mature T cells present. We use the same mathematical form (given in the appendix) for all such hypothesized effects of mature cells and PBL. This function is always scaled by a factor, $H_i^{jk}$ (with $i$ denoting the parameter that we are exerting the effect on, and $j$ and $k$ are indices that indicate whether the effect is exerted by syngeneic or allogeneic cells, on syngeneic or allogeneic cells). These factors take values between $-1$ and 1, to vary

TABLE V Simulations with PBL Effects on Thymocytes

| PBL seeded | Donor type of developing cells (%) | | | $RR_c$ | Cell numbers (× $10^4$) |
|---|---|---|---|---|---|
| | SBM | ABM | PBL | | |
| SYN[a] | 63 | 17 | 20 | 79 | 12 |
| SA | 53 | 27 | 20 | 67 | 12 |
| — | 54 | 46 | 0 | 54 | 12 |
| ALLO | 37 | 43 | 20 | 46 | 12 |
| SYN[b] | 40 | 9 | 51 | 81 | 8.0 |
| SA | 46 | 13 | 41 | 78 | 9.1 |
| — | 54 | 46 | 0 | 54 | 12.0 |
| ALLO | 43 | 37 | 20 | 54 | 12.0 |

[a]First set: Results of simulations with PBL affecting only newly seeded progenitors. Seeded: Mixtures of syngeneic and allogeneic $Thy1^-$ BM cells. Parameters and other details are as in Table IV, with the exception of $H_\alpha^{sa} = -1$ and $H_\alpha^{as} = -0.5$. SBM: Syngeneic BM-derived thymocytes. ABM: Allogeneic BM-derived thymocytes.

[b]Second set: Simulations with PBL affecting thymocyte proliferation. Seeded: Mixtures of syngeneic and allogeneic $Thy1^-$ BM cells. Parameters and other details are as in Table IV, with the exception of $H_{b_g}^{ss} = -0.4$ and $H_{b_g}^{sa} = -0.5$.

the direction and magnitude of the effect under consideration. Further details are given in the appendix. In the following simulations, we use the same parameters and initial conditions as in the previous simulations, and modify only the $H_i^{jk}$ factors denoting the feedback effects exerted by mature T cells.

### Effect of Mature Cells on Newly Seeded Progenitor Cells

For the sake of simplicity, we checked only one type of effect at a time, with the aim of identifying effects that may be worth checking experimentally. Even so, each effect—for example, on seeding, as in the preceding example—may in principle involve four parameters. Hence, for each effect, we performed simulations where the four parameters (e.g., $H_\alpha^{ss}$, $H_\alpha^{as}$, $H_\alpha^{sa}$, and $H_\alpha^{aa}$) were assigned the values $-1$, $-0.5$, 0.5, and 1. A finer tuning of parameter values was attempted in several cases after the "promising" direction was identified. The combinations of values that led to a successful reconstruction of experimental results are discussed in what follows.

The best results were obtained when we assumed that syngeneic mature T cells interfere with the seeding of allogeneic progenitors ($H_\alpha^{sa} = -1$), although using this modification alone still resulted in simulated compositions outside the experimental data ranges (results not shown). When we additionally assumed that allogeneic mature T cells also interfere with the seeding of syngeneic progenitors, though not to the same extent ($H_\alpha^{as} = -0.5$, the relative reconstitution ratios were completely within the range of the experimentally measured values (Table V). The fourth line in each simulation set in Table V gives the prediction of our model for an experiment with allogeneic PBL, under the same hypothesis. Similar results are obtained when assuming mature cells interfere not with seeding itself but with the cell divisions of newly seeded cells (i.e., setting ($H_{bn}^{as} = -0.5$ and ($H_{bn}^{sa} = -1$ in functions $\mathfrak{F}_{bn}^{s}$ and $\mathfrak{F}_{bn}^{a} = -1$).

Whereas these simulations reproduce the relative compositions of the reconstituted thymuses, they do not reproduce the reduction in cell numbers observed in all experiments in which PBL were added.

### Effect of Mature Cells on Thymocyte Proliferation

In contrast to the results of the previous simulations, total cell numbers and proportions can be changed dramatically if we assume that mature T cells (PBL in this case) negatively affect cell replication in the $G$ compartment (the proliferation rate $b$). Results that are almost compatible with experiment (Table V) are achieved with the assumption that only mature syngeneic T cells have an effect, that is, $H_{b_g}^{ss} = -0.4$, $H_{b_g}^{sa} = -0.5$, $H_{b_g}^{as} = 0$, and $H_{b_g}^{aa} = 0$. However, cell numbers are still not sufficiently reduced, and any parameter combination that results in sufficiently reduced total cell numbers leads to exaggerated proportions of PBL and different $RR_c$ values. Since we believe the data on the composition of the cultures are more reliable than the data on total cell numbers, we give more weight to parameter combinations that lead to reconstitution of cell proportions.

Combining the assumption that mature syngeneic T cells have a negative effect on the proliferation in the *G* compartment with the assumption that mature allogeneic T cells may exert similar effects, though weaker, or with the assumptions of effects on seeding discussed before, does not lead to better results (not shown). It seems that the negative effect on proliferation is necessary and sufficient to explain the SDP phenomena.

### Effect of Mature Cells on Thymocyte Death

When we assume that mature T cells enhance cell death only in the *N* (seeding) compartment—which is the less likely hypothesis—we can reconstruct the relative ratios, but not the reduction in total cell numbers. Assuming that mature T cells enhance cell death in the *G* (proliferation and differentiation) compartment, we obtain almost the same results as we obtained from the hypothesis of reduction of cell replication in this compartment (results not shown). We cannot exclude the possibility that the mechanisms responsible for MHC-linked SDP involve enhancement of thymocyte death, rather than interference with thymocyte replication.

In the experiments, when unseparated, instead of Thy1$^-$, BM cells were seeded in mixtures, syngeneic cells always took over ($RR_c > 80$) whether PBL were added or not. When only syngeneic or only allogeneic unseparated BM cells were seeded, the results were similar to those of Thy1$^-$ BM cell seeding. In simulations mimicking these experiments, the conclusions were similar to those of the simulations of Thy1$^-$ BM seeding (data not shown).

### Including Nonzero Differentiation

In all of the preceding simulations, we neglected the differentiation of BM-derived thymocytes into mature T cells (i.e., we took $\delta_g = 0$), assuming that it occurs at a very low rate and hence that the effects of mature cells will be small (relative to those of PBL). In order to check this assumption, we repeated the preceding simulations with $\delta_g = 0.001\ \text{hr}^{-1}$ (which is reasonable, since the rate of mature T-cell production in the thymus is $\leq 3\%\ \text{day}^{-1}$). The results of these simulations were not significantly different from the results of the previous simulations (data not shown), confirming our assertion that MHC-linked SDP in the FTOC is determined long before functional BM-derived mature T cells are generated.

### Model Predictions

We now use the model to predict results that would be obtained in situations that were not (as yet) checked experimentally, employing those hypotheses that gave a good reconstruction of known experimental results, that is, that mature T cells have a negative effect on thymocyte seeding or replication. In Table V, the fourth line of each simulation set gives our prediction for the case of mixture (syngeneic and allogeneic BM) seeding with the addition of allogeneic PBL. Note especially the fourth line in the second set: Under the same assumptions that lead to the best retrieval of experimental results, the model generates the prediction that allogeneic mature T cells will have a smaller effect than syngeneic mature T cells. This is in line with the conclusions of the unseparated BM seeding experiments, including sequential seeding experiments (Sharp et al., 1991).

### DISCUSSION

The goal of this study was to determine the most plausible mechanisms underlying the phenomenon of MHC-linked syngeneic developmental preference (SDP), that is, the advantage that syngeneic bone marrow (BM) cells have over allogeneic cells when colonizing the thymus under competitive conditions (Eren et al., 1989; Sharp et al., 1991, 1995; Fridkis-Hareli et al., 1993). Our strategy was to use mathematical modeling to screen various hypotheses that could explain MHC-linked SDP. We aimed at constructing the simplest mathematical model that incorporates our present knowledge concerning thymocyte development. Using various parameter combinations, we modeled the following alternative hypotheses: (a) Mature T cells in the thymus interact with thymocytes only through competition for the thymic stroma. This hypothesis takes an "ecological" view of the thymus as an environment in which thymic subpopulations are competing for limited resources. A similar view was used to show the role of competitive exclusion in the development of B-cell Freitas et al., 1995) and T-cell (DeBoer and Perelson, 1994) repertoires. In our case, however, we do not expect competitive exclusion, but rather that the system would approach a steady state in which thymic subpopulations are all present, because these subpopulations are in subsequent compartments in one differentiation pathway. (b) Mature T cells interfere with the seeding of all types of progenitor cells onto the thymus, based on the finding that early events in the seeding stage are crucial in determining thymic output (Sharp et al., 1990; Mehr et al., 1994, 1995), and that allogeneic mature T cells have a weaker effect than syngeneic mature T cells. (c) Syngeneic mature T cells interfere with thymocyte proliferation, or equivalently enhance thymocyte death; these effects are more pronounced when the progenitors are of allogeneic origin. This hypothesis was suggested by the observation that total cell numbers are mostly affected by cell proliferation following seeding (Mehr et al., 1996b).

Hypothesis (a) was not able to explain either the relative reconstitution ratios or the cell-number reductions observed in experiments with PBL (Table I). Hypothesis (b) was sufficient to explain the relative reconstitution ratios but not the cell-number reductions, and hypothesis (c) was sufficient to explain both observations. In simulations of sequential seeding experiments, competition in the newly seeded cell compartment was found to be crucial, and thus we cannot exclude the possibility that mature cells also negatively affect progenitor-cell seeding. However, the most important conclusion from the model is that the effect of mature syngeneic T cells is not exerted only at the seeding stage, as suggested by the sequential seeding experiments, but also at later stages of development, where proliferation and negative selection are important. Double-positive thymocytes are sensitive to signals inducing programmed cell death or blocking proliferation, and syngeneic mature T cells, activated by the presence of allogeneic thymocyte progenitors, could be the source of such signals.

We may speculate on possible mechanisms by which mature T cells would negatively affect progenitor-cell seeding or thymocyte proliferation. Mature T cells may physically interfere with thymic seeding if they can bind the same stromal cells that constitute the "seeding niches." This hypothesis cannot be directly examined because the identity of these niches has not yet been elucidated. Control of thymocyte proliferation (or death) may operate through cytokines secreted by mature T cells, possibly induced by the thymic stroma. For example, IFN-$\gamma$ is known to inhibit proliferation of Th2 cells *in vitro* (Fernandez-Botran et al., 1988; Gajewski and Fitch, 1988). The activity of functional T cells in the thymus should be monitored in order to examine this possibility.

In order to elucidate the mechanisms further, it is important to determine the exact identity of the mature T cells that induce MHC-linked syngeneic preference. So far, they have been identified as $CD3^+Thy1^+$ (Sharp et al., 1995). Experiments in which only $CD4^+CD8^-$ or only $CD4^-CD8^+$ were added to the FT-BM cocultures have recently been performed using syngeneic combinations (Fridkis-Hareli et al., 1994). The combination of these experiments and mathematical analysis of their results (Mehr et al., 1996a, 1996b) suggested that in purely syngeneic systems, mature T cells may interfere with the seeding of all types of progenitor cells, and interfere with the subsequent proliferation of thymocytes. This analysis suggested that thymocyte development is subject to regulation through two feedback loops: $CD4^+$ cells exert a negative feedback on the double-positive compartment, and a positive feedback on the $CD4^+$ single-positive compartment. The relevance of these findings for HIV pathogenesis was also studied (Mehr and Perelson, 1997). The observation that SDP is mediated, to a great extent, by MHC class II yet also by MHC class I molecules (Sharp et al., 1995) suggests that the first event of competition for seeding of the progenitors in the thymus may be controlled by $CD4^+$ cells, via an MHC class II recognition event, whereas the subsequent effects on the DP subset are mediated by $CD8^+$ cells. Indeed, preliminary data indicate that $CD8^+$ cells may play a role at this later stage of allogeneic cell development in the thymus (data not published). Thus, in addition to directing late-stage selection processes, MHC molecules may also play a role in the very early events that occur on the first entry into the thymus of BM-derived T-cell progenitors, which do not yet express the TCR or functional T-cell markers.

Mature T cells present in the thymus may be derived from maturing thymocytes, or consist of recirculating peripheral T cells that reenter the thymus (Hirokawa et al., 1989). In the neonate, the cells that reenter may be resting or activated T cells (Surh et al., 1993), whereas in the adult, reentry seems to be restricted to activated T cells (Fink et al., 1984; Agus et al., 1991), especially when the thymus is infected (Gossmann et al., 1991). Our results suggest that the presence of mature T cells in the thymus, whether these cells derive from thymocytes or peripheral T cells, has significance in the regulation of thymocyte development.

### APPENDIX: DETAILS OF THE MATHEMATICAL MODEL

We first derive the equations of our model for a one-cell population. Competition for seeding niches (Mehr et al., 1994) is expressed by a crowding term $(1 - N/K_n)$, which multiplies both the seeding and proliferation rates. $K_n$ is the carrying capacity of the seeding compartment. The rate of seeding per niche is denoted by $\alpha$, so that the number of cells seeded into the thymus in the first hour is $\alpha K_n$; the value of $\alpha$ can be calculated from the known fraction of T-cell progenitors in the BM. The cell division rate of newly seeded and developing thymocytes, denoted by $b$, can be calculated from the cell cycle time of thymocytes (24-40 hr, depending on the system studied; Baron and Penit, 1990). Newly seeded cells differentiate into the growing thymocyte compartment with rate $\delta_n$. Cell death is neglected in this compartment (Baron and Penit, 1990; Egerton et al., 1990). Thus, the number of cells in the newly seeded cell compartment, $N$, changes with time according to

$$\frac{dN}{dt} = (\alpha K_n + bN)\left(1 - \frac{N}{K_n}\right) - \delta_n N \qquad (3)$$

The equation for the growth compartment ($G$) is similar, except for the following differences. The entry of cells into this compartment from the previous one is given by $\delta_n N$. Since we assume that the proliferation rate of the cells in this compartment may be dependent on whether they are derived from syngeneic or allogeneic BM, we modify $b$ in the equation for $G$ by $(1 + \epsilon)$. The parameter $\epsilon$ may take different values, representing the difference in stromal support for syngeneic and allogeneic thymocytes. Additionally, these cells may become mature, functional T cells at rate $\delta_g$ ($\delta_g$ is very small—less than 3% per day; (Shortman et al., 1990), or die at rate $\mu_g$, which may be rather large due to thymic selection. These cells compete with the mature cells for the thymic stroma; hence, there is a crowding term with $K$, the total thymic carrying capacity, limiting cell proliferation in these compartments. Thus, the number of cells in the $G$ compartment changes according to

$$\frac{dG}{dt} = \delta_n N + (1 + \epsilon)bG\left(1 - \frac{G + M + P}{K}\right) - \mu_g G - \delta_g G \qquad (4)$$

Similar considerations lead to equations for the time evolution of BM-derived ($M$) and PBL-derived ($P$) mature T cells:

$$\frac{dM}{dt} = \delta_g G + (1 + \epsilon)b_m M\left(1 - \frac{G + M + P}{K}\right) - \mu_m M \qquad (5)$$

$$\frac{dP}{dt} = (1 + \epsilon)b_m P\left(1 - \frac{G + M + P}{K}\right) - \mu_m P \qquad (6)$$

Our findings indicate that it is reasonable to take $b_m \ll b$ and $\mu_m = 0$ in our modeling. In this range of parameters, Eqs. (3) to (6) have a stable steady state with $0 < N < K_n$ and $0 < G + M + P < K$, that is, the values are biologically reasonable.

Generalizing our model for two cell types (syngeneic and allogeneic) competing in the same thymus is now straightforward (Figure 2). We denote by $N_s$, $G_s$, $M_s$, and $P_s$ the four compartments of syngeneic cells, and by $N_a$, $G_a$, $M_a$, and $P_a$ the corresponding allogeneic cell compartments. Similar subscripts denote parameter values that are assumed to be different for cells of the two sources, and the resulting eight equations are similar to the equations of the one-population model, except that $(1 - N/K_n)$ in the equations for $N_s$ and $N_a$ is replaced by $[1 - (N_a + N_a)/K_n]$, and $(G + M + P)$ in the other equations is replaced by $T_m = G_s + M_s + P_s + G_a + M_a + P_a + P_{sa}$, the total number of mature cells of all sources (PBL and thymocytes, syngeneic and allogeneic, and semiallogeneic as defined in the next paragraph).

We make the simplifying assumption that half of the mature T cells in semiallogeneic PBL have TCRs restricted to syngeneic MHC types, and the other half have TCRs restricted to allogeneic MHC types. Similarly, we assume that each cell in the PBL expresses on its surface MHC molecules, of which half are of the syngeneic type and half of the allogeneic type. We denote the number of these cells by $P_{sa}$[Eq. (7)].

$$\frac{dP_{sa}}{dt} = \left(1 + \frac{\epsilon_a + \epsilon_s}{2}\right) b_m P_{sa} C_m - \mu_m P_{sa} \qquad (7)$$

As long as we do not add the modification of parameters due to effects of mature T cells, both the syngeneic and allogeneic populations have almost identical parameters, except $\epsilon$. Hence, the final ratio between $N_s$ and $N_a$ is mostly determined by the results of the seeding stage (cell numbers at $t = 72$ or $t = 96$ hr). In particular, the population that is seeded first has a significant advantage (Figure 3a and 3b).

To include the various possible interactions between cells from the syngeneic and allogeneic populations, we generalized the two-population equations further. Each hypothesized effect can be represented as a modification of one or more of the parameters. For example, in order to model an effect of mature T cells on the rate of seeding of syngeneic progenitors, we multiplied the seeding term, $\alpha_s K_n = [1 - (N_s + N_a)/K_n]$, by a function

$$\mathfrak{F}_\alpha^s \equiv 1 + H_\alpha^{ss} \frac{M_s + P_s + \frac{1}{2} P_{sa}}{M_s + P_s + \frac{1}{2} P_{sa} + K_{m_s}} + H_\alpha^{as} \frac{M_a + P_a + \frac{1}{2} P_{sa}}{M_a + P_a + \frac{1}{2} P_{sa} + K_{m_a}} \qquad (8)$$

The notation $\mathfrak{F}_\alpha{}^s$ indicates that the effect is exerted on syngeneic cells through modification of $\alpha$. $H_\alpha{}^{jk}$ are parameters that modify $\alpha$. The superscript $(jk)$ indicates the effect of $j$-type cells on $k$-type cells; $j$ and $k$ take the values $n$ (syngeneic) or $a$ (allogeneic). The $H$ parameters take values between $-1$ and 1, because the observed changes in thymic composition indicate that a change in parameter values of more than twofold is probably not required. The quantities $K_{m_s}$ and $K_{m_a}$ are the dose of seeded PBL at which half the maximum SDP is obtained; it is about 10% of the inoculum, or $K_{m_a} = 6000$ cells, for allogeneic T cells, and between 2.5-5%, or $K_{m_s} = 1500$-$3000$ cells, for syngeneic T cells (data not published).

We use the same functional form for all hypothesized effects of mature cells and PBL, and use similar $H_i^{jk}$ parameters, that have values between $-1$ and 1, to vary the direction and magnitude of the effects. In addition to $H_\alpha^{jk}$, the magnitude of the effect of mature cells on seeding, we use $H_{b_n}^{jk}$, $H_{b_g}^{jk}$, and $H_{b_m}^{jk}$, the magnitude of the effect of mature cells on the proliferation of cells in the seeding, differentiation, and mature cell compartments, respectively; $H_{\delta_n}^{jk}$, the magnitude of the effect of mature cells on differentiation of seeded cells; and $H_\lambda^{jk}$, the magnitude of the effect of mature cells on thymocyte death in the two immature cell compartments, where $\lambda$ is the rate of death in the corresponding compartment that is

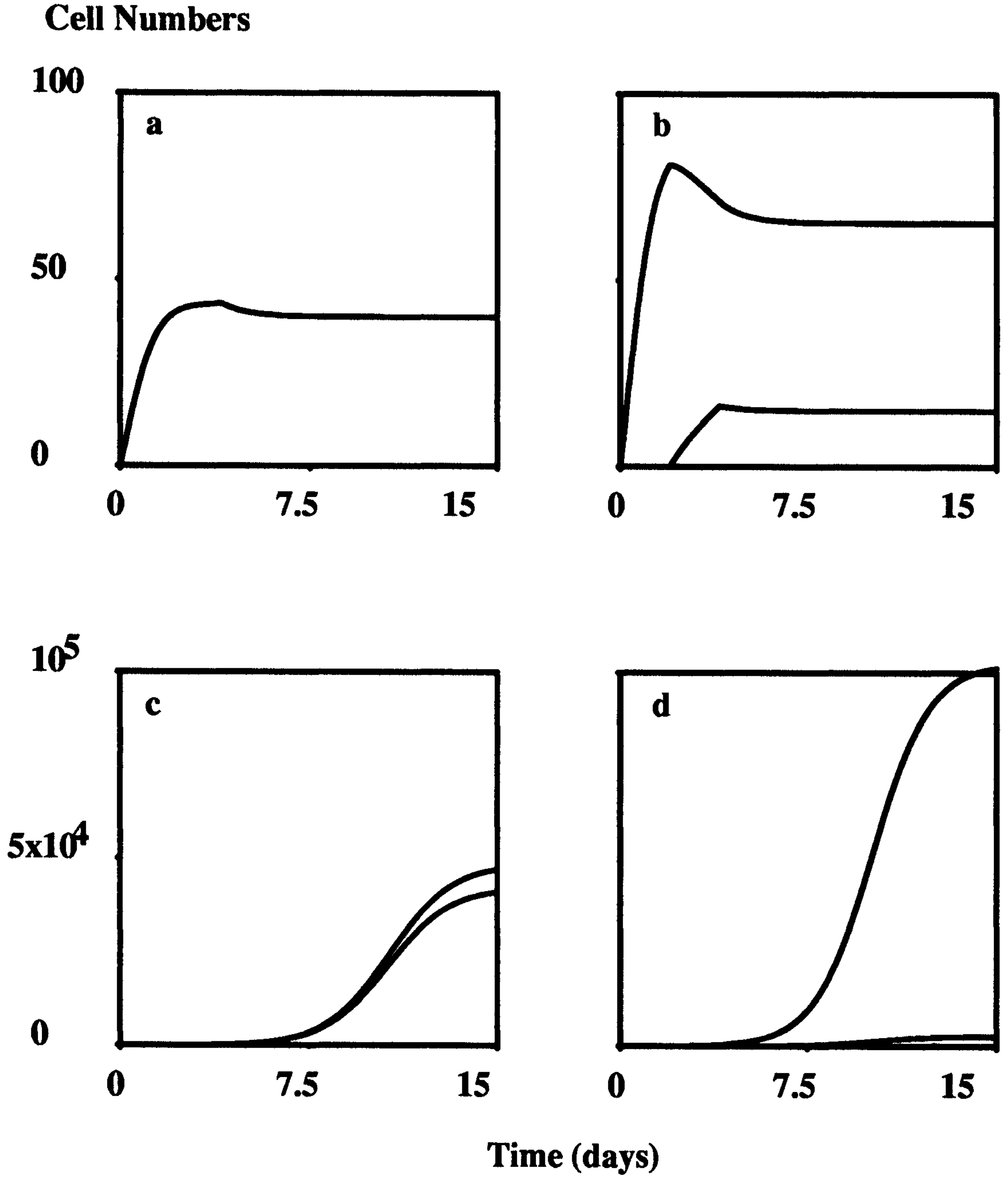

FIGURE 3 (**a** and **b**) Numbers of cells in the seeding compartment in simulations of the two-population model, with the parameters and initial conditions given in Table II, for the case of unsorted BM seeding without PBL. (a) Mixture seeding (1:1). The curves for the two populations coincide. (b) Sequential seeding, where syngeneic BM are seeded first. The upper curve represents syngeneic cells, and the lower allogeneic cells. It is evident that, although in this compartment both syngeneic and allogeneic cells have the same parameters, the population that is seeded first gains a significant advantage. (**c** and **d**) Numbers of cells in the proliferation compartment in simulations of mixture (c) and sequential (d) seeding. In both cases, the upper curve represents syngeneic cells and the lower curve represents allogeneic cells. Both populations approach steady-state values after 15 days.

caused by mature cells (which may be different than that of the "default" cell death denoted by $\mu_g$). In simulations reported earlier, we examined the effect of each interaction separately, or at most combinations of two effects. Hence, in each simulation, all the $H_i^{jk}$, parameters except one or two are set to zero.

### Acknowledgments

Simulations were performed using "GRIND" software (© 1983, R. J. De Boer). Most of this work was performed under the auspices of the U.S. Department of Energy and supported by NIH Grants AI28433 and RR06555. It was also supported by the U.S.-Israel Binational Science Foundation Grant 92-00171; and the Santa Fe Institute through a Joseph P. and Jeanne M. Sullivan Foundation grant to their Theoretical Immunology program.

### References

Agus D.B., Surh C.D., and Sprent J. (1991). Reentry of T cells to the adult thymus is restricted to activated T cells. J. Exp. Med. **173**:1039-1046.

Baron C., and Penit C. (1990). Study of the thymocyte cell cycle by bivariate analysis of incorporated bromodeoxyuridine and DNA content. Eur. J. Immunol. **20**:1231-1236.

Blackman M., Kappler J., and Marrack P. (1990). The role of the T cell receptor in positive and negative selection of developing T cells. Science **248**:1335-1341.

DeBoer R.J., and Perelson A. (1994). T cell repertoires and competitive exclusion. J. Theor. Biol. **169**:375-390.

Egerton M., Scollay R., and Shortman K. (1990). Kinetics of mature T-cell development in the thymus. Proc. Natl. Acad. Sci. USA **87**:2579-2582.

Eren R., Abel L., and Globerson A. (1989). Syngeneic preference manifested by thymic stroma during development of thymocytes from bone marrow cells. Eur. J. Immunol. **19**:2087-2092.

Eren R., Globerson A., Abel L., and Zharhary D. (1990). Quantitative analysis of bone marrow thymic progenitors in young and aged mice. Cell. Immunol. **127**:238-246.

Fernandez-Botran R., Sanders V.M., Mosmann T.R., and Vitetta E.S. (1988). Lymphokine-mediated regulation of the proliferative response of clones of T helper 1 and T helper 2 cells. J. Exp. Med. **168**:543-558.

Fink P.J., Bevan M.J., and Weissman I.L. (1984). Thymic cytotoxic T lymphocytes are primed in vivo to minor histocompatibility antigens. J. Exp. Med. **159**:436-451.

Francz P., Fridkis-Hareli M., Abel L., Bayreuther K., and Globerson A. (1992). Differential expression of thymic stromal membranal polypeptides in cocultures of fetal thymus and bone marrow cells from young and old mice. Mech. Ageing Dev. **64**:99-109.

Fridkis-Hareli M., Eren R., Sharp A., Abel L., Kukulansky T., and Globerson A. (1993). MHC recognition in colonization of the thymus by bone marrow cells. Cell. Immunol. **149**:91-98.

Fridkis-Hareli M., Mehr R., Abel L., and Globerson A. (1994). Developmental interactions of CD4+ T cells and thymocytes: Age-related differential effects. Mech. Ageing Dev. **73**:169-178.

Frietas A.A., Rosado M.M., Viale A.C., and Grandien A. (1995). The role of cellular competition in B cell survival and selection of B cell repertoires. Eur. J. Immunol. **25**:1729-1738.

Gajewski T.F., and Fitch F.W. (1988). Anti-proliferative effect of IFN-gamma in immune regulation. I. IFN-gamma inhibits the proliferation of Th2 but not Th1 murine helper T lymphocyte clones. J. Immunol. **130**:4225-4252.

Gossmann J., Lohler J., and Lehmann-Grube F. (1991). Entry of antivirally active T lymphocytes into the thymus of virus-infected mice. J. Immunol. **146**:293-297.

Hirokawa K., Utsuyama M., and Sado T. (1989). Immunohistological analysis of immigration of thymocyte-precursors into the thymus: Evidence for immigration of peripheral T cells into the thymic medulla. Cell. Immunol. **119**:160-170.

Kisielow P., and von Boehmer H. (1990). Negative and positive selection of immature thymocytes: Timing and the role of the ligand for $\alpha$-$\beta$ T cell receptor. Semin. Immunol. **2**:35-44.

Mehr R., Abel L., Ubezio P., Globerson A., and Agur Z. (1993). A mathematical model of the effect of aging on bone marrow cells colonizing the thymus. Mech. Ageing Dev. **67**:159-172.

Mehr R., Fridkis-Hareli M., Abel L., Segel L., and Globerson A. (1995). Lymphocyte development in irradiated thymuses: Dynamics of colonization by progenitor cells and regeneration of resident cells. J. Theor. Biol. **177**:181-192.

Mehr R., and Perelson A. (1997). Blind homeostasis and the CD4:CD8 ratio in the thymus and peripheral blood. J. AIDS Human Retrovirol. **14**:387–398.

Mehr R., Perelson A., Fridkis-Hareli M., and Globerson A. (1996a). Feedback regulation of T cell development: Manifestations in aging. Mech. Ageing Dev. **91**:195-210.

Mehr R., Perelson A., Fridkis-Hareli M., and Globerson A. (1996b). Feedback regulation of T cell development in the thymus. J. Theor. Biol. **181**:157-167.

Mehr R., Segel L., Sharp A., and Globerson A. (1994). Colonization of the thymus by T cell progenitors: Models for cell-cell interactions. J. Theor. Biol. **170**:247-257.

Metcalf D. (1963). The autonomous behaviour of normal thymus grafts. Austral. J. Exp. Biol. **41**:437-448.

Reisner Y., and Martelli M.F. (1995). Bone marrow transplantation across HLA barriers by increasing the number of transplanted cells. Immunol. Today **16**:437-440.

Sharp A., Fridkis-Hareli M., Eren R., Kukulansky T., Abel L., and Globerson A. (1995). MHC-linked colonization of the thymus and thymocyte development: Effects of mature T lymphocytes. Intl. Arch. Allergy Immunol. **106**:13-19.

Sharp A., Kukulansky T., and Globerson A. (1990). *In vitro* analysis of age-related changes in the developmental potential of bone marrow thymocyte progenitors. Eur. J. Immunol. **20**:2541-2546.

Sharp A., Kukulansky T., and Globerson A. (1991). Interactions of bone marrow cells from young and old mice with syngeneic and allogeneic thymic tissue. Cell. Immunol. **138**:280-288.

Shortman K., Egerton M., Spangrude G., and Scollay R. (1990). The generation and fate of thymocytes. Semin. Immunol. **2**:3-12.

Surh C.D., Sprent J., and Webb S.R. (1993). Exclusion of circulating T cells from the thymus does not apply in the neonatal period. J. Exp. Med. **177**:379-385.

Von Boehmer H. (1991). Positive and negative selection of the $\alpha$-$\beta$ T-cell repertoire *in vivo*. Curr. Opin. Immunol. **3**:210-215.

Von Boehmer H., and Kisielow P. (1990). Self-nonself discrimination by T cells. Science **248**:1369-1373.

